# Central retinal vein occlusion in patients with metastatic solid tumors on tyrosine kinase inhibitors: a report of case series and literature review

**DOI:** 10.3389/fmed.2024.1362108

**Published:** 2024-06-20

**Authors:** Mingyue Luo, Lu Sun, Rongping Dai, Youxin Chen, Chan Wu

**Affiliations:** ^1^Department of Ophthalmology, Peking Union Medical College Hospital, Chinese Academy of Medical Sciences, Beijing, China; ^2^Key Laboratory of Ocular Fundus Diseases, Chinese Academy of Medical Sciences, Beijing, China

**Keywords:** retinal vein occlusion, macular edema, tyrosine kinase inhibitors, metastasis, intravitreal dexamethasone implant

## Abstract

**Background:**

Central retinal vein occlusion (CRVO) is a rare adverse effect related to the use of tyrosine kinase inhibitors (TKIs) in patients with metastatic malignancies, which has only been reported in several case reports.

**Case presentation:**

We reported the case series of three CRVO patients on regular regimens of TKIs as part of targeted therapies for metastatic malignancies, all of whom were otherwise healthy with no or well-controlled systemic conditions. All these patients received injections of intravitreal dexamethasone implant (IDI) and achieved a fluid-free macula at the end of the visit. In addition, we reviewed the existing literature on this subject and present here an updated analysis of the related TKIs, ocular presentation, treatment, and prognosis.

**Conclusion:**

All patients diagnosed with CRVO on TKIs received dexamethasone implant treatment and obtained a fluid-free macula. We would like to raise awareness among our colleague oncologists about the possibility of CRVO related to TKI use and the necessity for patients to be screened regularly by a retinal specialist.

## Introduction

Retinal vein occlusions (RVOs) are a group of vision-threatening disorders with the second-highest rate of retinal vascular blindness after diabetic retinopathy (1) and a worldwide prevalence of approximately 0.1–0.5% (1–3). Those whose obstruction occurs before the major bifurcation of the central retinal vein fall into the clinical entity of central retinal vein occlusions (CRVOs), characterized by engorgement and tortuosity of the retinal veins in all quadrants with retinal hemorrhages, cotton wool spots (CWSs), swelling of the optic disc, and macular edema (ME) (4). Instead of venous compression at the arteriovenous crossing site often observed in branch RVOs (5), thrombosis of the central retinal vein posterior to the lamina cribrosa is typically the cause of CRVO (6).

Advancing age, hyperlipidemia, hypertension (HTN), and primary open-angle glaucoma are well-established systemic and ocular risk factors for CRVO, while diabetes mellitus (DM) is less so (6, 7). Other conditions are less commonly associated with CRVO, including sleep apnea, homocystinuria, stroke, and thrombophilia, which should be given special attention, especially for younger patients (8, 9).

CRVO, though uncommon, is recognized as a severe adverse event related to the use of targeted anti-cancer drugs, especially for mitogen-activated protein kinase kinase (MEK) and human gene that encodes a protein (BRAF) inhibitors, usually for the treatment of metastatic melanoma (10, 11). It is even more rarely reported in the use of tyrosine kinase inhibitors (TKIs) and fibroblast growth factor receptor (FGFR) inhibitors (12), providing sporadic evidence for the retinal toxicity of these drugs. Herein, we report the case series of three CRVO patients on regular regimens of TKIs as part of targeted therapies for metastatic malignancies, all of whom were otherwise healthy with no or well-controlled systemic conditions.

## Case series presentation

### Case 1

**Figure 1:**
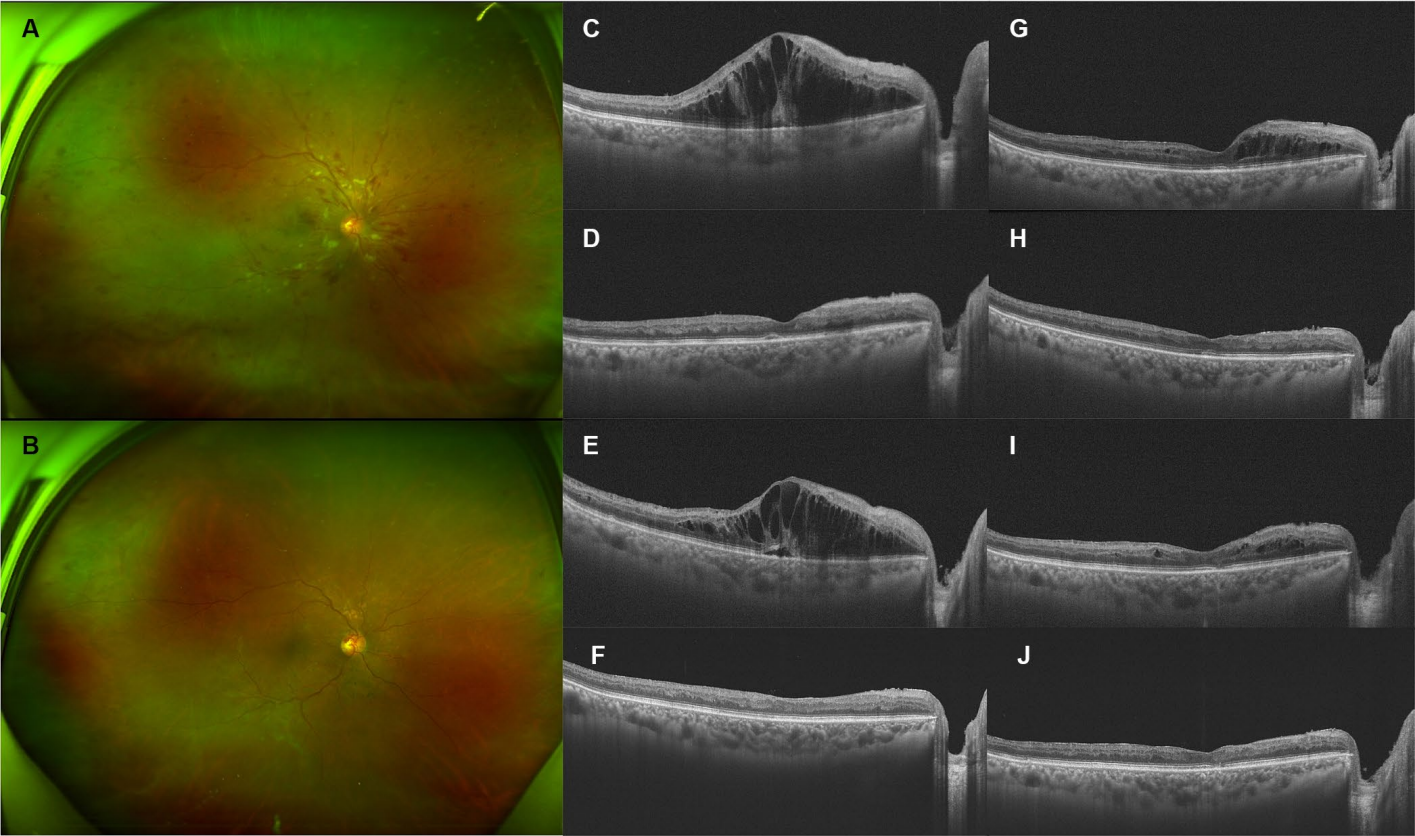
Retinal and optical coherence tomography (OCT) images of patient 1. **(A,B)** Before and after four injections of intravitreal dexamethasone implant (IDI). **(C)** Before the first injection. **(D)** One month after the first injection, the macular edema (ME) completely resolved. **(E)** Three months after the first injection, the ME recurred, and the second injection was prescribed. **(F)** Two months after the second injection, the ME completely resolved. **(G)** Three months after the second injection, ME recurred, and the third injection was prescribed. **(H)** Two months after the third injection, the ME completely resolved. **(I)** Three months after the third injection, macular injection recurred, and the fourth injection was prescribed. **(J)** OCT image of the last visit. The macula remained fluid-free for nine months after the fourth injection, until the last visit.

A 65-year-old man presented with suddenly decreased vision in his right eye for 20 days. He was on a regular regimen of sorafenib 400 mg twice a day for metastatic renal cell carcinoma (RCC) for more than 2 years after left radical nephrectomy. No ocular or systemic diseases or systemic medications were reported. On physical examination, the visual acuity (VA) of the right eye was counting fingers. The intraocular pressure (IOP) of the right eye was recorded at 14 mmHg. A mild cataract was noticed. Funduscopic examination, fundus photographs, and optical coherence tomography (OCT) B-scan (Figure 1) showed severe ME with tortuous retinal veins, scattered retinal hemorrhagic, and CWSs in the posterior pole with no obvious non-perfusion area or neovascularization. The diagnosis of CRVO of the right eye was made. Homocysteine, protein C and S, activated protein C, and antithrombase levels were screened for systemic risk factors of CRVO and thrombophilia, which were within the normal range. Long-term use of sorafenib was considered the probable major cause of CRVO after ruling out other risk factors. Injection of sustained-release intravitreal dexamethasone implant (IDI, Ozurdex, Allergan) was prescribed. One month after the injection, ME of the right eye completely resolved but recurred at the next visit 2 months later. In total, he received three more injections, at a 3-month interval, due to repeated absorption and recurrence of ME, which became less severe than the previous recurrence. After four injections, the macula remained fluid-free until the last visit 9 months later, with his VA improving to 20/133.

### Case 2

**Figure 2:**
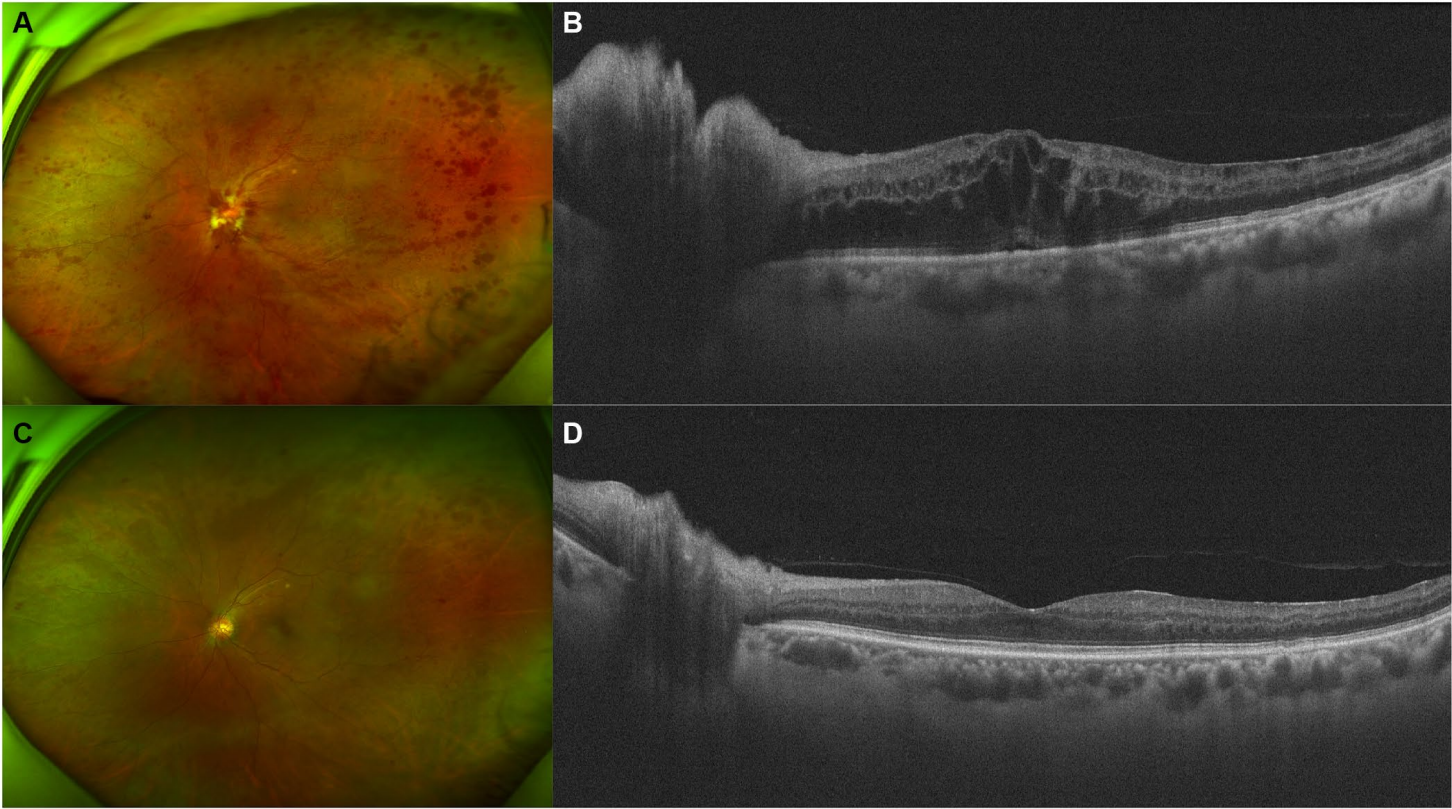
Retinal and optical coherence tomography (OCT) images of patient 2. **(A,B)** Retinal and OCT images on presentation. Flame-like disc hemorrhages as well as “dot and blot” hemorrhages over the whole retina were obvious. OCT demonstrated severe macular and disc edema. **(C,D)** Five months after injection of intravitreal dexamethasone implant (IDI). Most of the hemorrhages and the macular edema resolved.

A 66-year-old man presented with decreased VA and metamorphopsia in his left eye for 2 weeks. He had been on sorafenib 400 mg twice a day for metastatic RCC for 4 years, before which he had left radical nephrectomy 3 years ago. No systemic comorbidities or other systemic medications were reported. Upon initial examination, the VA of his left eye was measured at 20/200, and the IOP of the left eye was recorded at 16 mmHg. On physical examination, optic disc edema, flame-like disc hemorrhages, “dot and blot” hemorrhages, and ME were noticed (Figure 2), and the diagnosis of CRVO of the left eye was made. Hyperhomocysteinemia and thrombophilia were screened and excluded. After one injection of IDI, his VA improved to 20/40, and photocoagulation of the non-perfusion area in the temporal retina was demonstrated by OCT angiography, after which he lost follow-up due to the coronavirus disease 2019 (COVID-19) pandemic.

### Case 3

**Figure 3:**
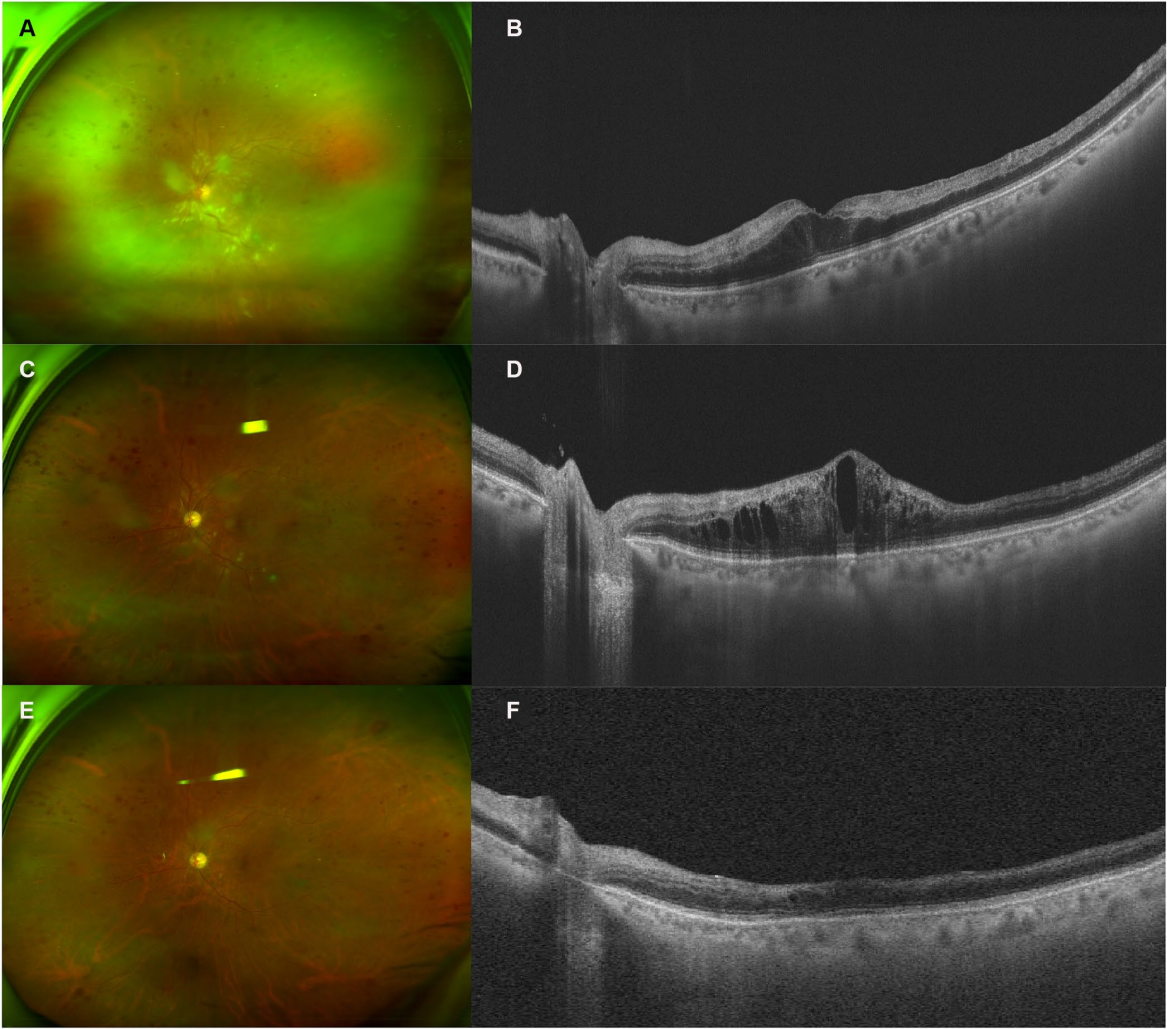
Retinal and optical coherence tomography (OCT) images of patient 3. **(A,B)** Retinal and OCT images on presentation. Notice the edematous disc, scattered cotton wool spots (CWSs) in the posterior pole, and “dot and blot” retinal hemorrhages. The white retinal vessels in the periphery indicated extensive retinal ischemia, corresponding to the hyper-reflective band spanning the inner nuclear layer (INL). **(C,D)** Two months after the intravitreal injection of ranibizumab, the CWS and hemorrhage were partially absorbed. However, OCT demonstrated a recurrence of macular edema (ME). **(E,F)** One month after the injection of intravitreal dexamethasone implant (IDI), most CWSs and ME resolved, with some remnant of retinal hemorrhages. INL atrophy could be observed.

A 68-year-old man presented with decreased vision in his left eye for 1 week. He had been on a regular dose of apatinib 250 mg once a day and camrelizumab 3 mg/kg every 2 weeks for metastatic thyroid cancer for 1 year before discontinuing the camrelizumab therapy 1 week ago due to abnormal liver function. He was diagnosed with HTN for over 10 years, for which he took nifedipine 30 mg once a day and valsartan 80 mg three times a day. His blood pressure (BP) was 128/79 mmHg at presentation, with a self-reported BP range over the previous 2-year period of 90–130/65–85 mmHg. He was diagnosed with DM for 1 year. He took metformin hydrochloride 0.5 g three times a day with a self-reported fasting blood glucose (BG) range of 4–5.5 mmol/L and a postprandial BG range of 6.5–7.5 mmol/L. In addition, he used levothyroxine sodium 175 μg once a day as postoperative treatment. On presentation, the VA of the left eye was 20/2000, compared to 20/60 during his last consultation for a cataract 2 months ago. The IOP of the left eye was 13 mmHg. On fundus examination, the disc was edematous, with scattered CWSs and “dot and blot” hemorrhages of the posterior pole and a grayish edematous retina in the upper nasal quadrant (Figure 3). The diagnosis of CRVO and retinal artery occlusion of the left eye was made. Hyperhomocysteinemia and thrombophilia were also screened and excluded. He was prescribed one intravitreal injection of ranibizumab, after which the ME did not resolve. Therefore, another injection of IDI was prescribed. One month after the injection, the ME improved significantly, and subsequent PRP was finished. OCT showed that the retinal condition was stable, despite little improvement of his VA. The patient lost follow-up afterward due to the COVID-19 pandemic.

## Discussion

TKIs function by inhibiting corresponding kinases from catalyzing downstream phosphorylation (13). Since being approved in 2001 by the Food and Drug Administration (FDA) of the United States, TKIs have greatly improved the life expectancy of numerous cancer patients. They are highly potent in stabilizing tumor progression with fewer side effects compared to cytotoxic chemotherapeutic agents (14). Currently, TKIs are designed to target multiple pathways for cumulative antitumor efficacy. Although most TKIs are designed to be highly selective, other targets can be covered unexpectedly (14), which partially explains the ocular side effects of targeted therapy (15).

Cases reporting CRVO and other retinal vascular abnormalities related to the use of TKIs are summarized in Table 1 (16–21). Since 2012, several cases have been reported sporadically, four of which were CRVO. Of these cases, two were related to the use of sorafenib (16, 17), three were related to axitinib (19–21) and one was related to regorafenib (18). Interestingly, the TKIs used in previously reported cases (Table 1) and in our patients all belong to vascular endothelial growth factor receptor (VEGFR)-associated multitargeted TKIs (22), which target multiple receptors of endothelial cells, including VEGFR, platelet-derived growth factor receptor, FGFR, c-Kit, and c-Met. These TKIs block the kinase activity and the downstream transduction pathways involving angiogenesis, proliferation, migration, and cell survival (23), significantly improving the overall survival of some types of solid malignancies, including RCC and hepatocellular carcinoma. Some of these pathways, including the VEGF pathways, are shared by normal retinal tissues, contributing to retinal toxicity. Agents targeting VEGF pathways have been reported to increase the risk of ocular and systemic thromboembolic events (TEs), partially contributed by the dysregulation of endothelial cells and a decrease in prostaglandin-I 2 (PG-I 2) and nitric oxide (NO) (24). On the other hand, with endothelial cell death, exposure to phospholipid and extracellular matrix increases the tendency for thrombosis (25).

**Table 1:** Previous cases of retinal vein occlusion and retinal vascular abnormalities related to the use of tyrosine kinase inhibitors.

Authors, year	Age, sex	Systemic comorbidities	Primary cancer (metastasis)	Oral anti-cancer drug regimen before ocular symptoms	Ocular presentation	Systemic Drug regimen afterward	Ocular intervention	Ocular prognosis	Ref
Szczepanik et al., 2012	73, M	Previous hypovolemic shock	RCC (abdominal cavity, lung, neck, and mediastinal LN)	Sorafenib 200 mg BID*19 m	Bilateral CRVO	Enoxaparin 100 mg subcutaneously QD *5d → 60 mg QD*6 m	Laser photocoagulation OU and trans-scleral cryoapplication OD	VA 0.01 OD, 0.06 OS, NPA number decreased, persistent ME in the right eye	(16)
Li et al., 2016	42, M	None	Mixed RCC (lungs)	Sorafenib 800 mg QD *17 m	Right CRVO	Sorafenib stopped. Mecobalamine	None	Retinal NV, VA 0.4 OD.	(17)
Schvartsman et al., 2016	74, M	Well-controlled HTN, T2DM, hyperlipidemia, glaucoma	GIST (omentum)	Regorafenib 120 mg QD *8w → 80 mg QD*11 m	Right CRVO	Regorafenib switched to imatinib 800 mg QD	IV-bevacizumab	VA improved.	(18)
Kimura et al., 2019	57, M	HTN, hypothyroidism, hyperuricemia	RCC (lung, adrenal gland, thigh adductor muscle, liver, and brain)	Axitinib 10 mg QD * 5 m	Soft exudates OD	Axitinib switched to temsirolimus	None	VA decreased to 20/63	(19)
Jenkins et al., 2021	62, F	None	RCC (NA)	Axitinib 4 mg QD*4 m → 8 mg QD *2 m	Bilateral CWSs, RH	Axitinib 6 mg*3 m → biweekly nivolumab	None	Marked improvement, resolution of all CWS.	(20)
Pyare et al., 2022	65, M	Cerebrovascular accident, right hemiparesis	RCC (brain)	Sunitinib 50 mg QD*2y →Axitinib 5 mg BID*22 m	Bilateral CRVO	Stopped	IDI	NA	(21)

HTN, hypertension; T2DM, type 2 diabetes mellitus; RCC, renal cell carcinoma; LN, lymph node; GIST, gastrointestinal stromal tumor; NA, not available; CRVO, central retinal vein occlusion; CWS, cotton wool spot; RH, retinal hemorrhage; IV, intravitreal; IDI, intravitreal dexamethasone implant; VA, visual acuity; ME, macular edema; NPA, non-perfusion area; NV, neovascularization.

It may seem paradoxical to relate RVO to the systemic use of anti-VEGF agents, as intravitreal injection of anti-VEGF agents is the first-line therapy for RVO. In fact, local vascular toxicity has been observed with intravitreal injections. In patients with diabetic retinopathy, anti-VEGF agents showed enlargement of the foveal avascular zone, with no efficacy in improving retinal perfusion in some studies (26, 27). In patients with RVO, aggravation of retinal perfusion and development of the new non-perfusion area and CWSs after intravitreal injections were reported (28, 29). In addition, side effects of the posterior segment, such as RVO (18), ME, and central serous retinopathy, have been reported with systemic anti-VEGF agents (30), which are consistent with the results of animal experiments showing decreased local blood flow secondary to retinal vascular endothelial cell dysfunction with systemic anti-VEGF treatment (31). Bevacizumab, a commonly used anti-VEGF agent in systemic chemotherapy, is also the first generation of anti-VEGF agents for intravitreal injections. It increases the risk of venous thromboembolism development in cancer patients (32), and RVO is reported as an adverse effect when combined with chemotherapy for metastatic colorectal cancer (33). Although the underlying mechanism has not been fully elucidated, these observations justify the causal relationship between anti-VEGF agents and RVO.

Theoretically, hypercoagulation and thrombosis, which are commonly observed in patients with malignancies (34), seem to increase the risk of CRVO. However, up to now, the direct causal relationship between malignancies and CRVO was not well-established (35), and the results of previous studies were not consistent. The study by Kim et al. (36) identified an increased risk of subsequent cancer in patients with RVO. In other studies, thrombosis of retinal veins was not identified as a significant clinical marker for occult cancer (37), and RVO did not show an increased frequency of thrombophilia (38). The study by Toft-Petersen et al. (39) established the association between cancer and RVO, but the authors stated that the relationship reflects common risk factors instead of causal relationships. In addition, primary malignancy may trigger the elevation of anticardiolipin and antiphospholipid, resulting in RVO (40).

In fact, TEs related to the use of TKIs in other systems are not scarce. Cardiotoxicity is a known adverse effect of TKIs targeting VEGFR (41). Pantaleo et al. (42) reported coronary artery stenosis in a 58-year-old man on regular sorafenib for metastatic RCC (mRCC) for 2 years, whose cardiac function was normal and with no common cardiovascular risk factors. Axitinib was reported in relation to the cerebrovascular accident (CVA) (21, 43) and pulmonary embolism (43). The exact mechanism of TKI-related endothelial dysfunction has not been elucidated, yet due to the vital role of VEGF signaling pathways in the maintenance of retinal endothelial cell homeostasis and function, it is a fair guess that blockade of VEGF results in abnormal vascular integrity, which may increase the tendency for embolisms. Additionally, animal studies of sunitinib-related cardiac side effects suggest the impairment of mitochondrial function and the compromise of cellular energy homeostasis might be involved in TKI-related toxicity (41).

It should be extremely cautious to establish the cause of CRVO with targeted therapy. An important reason is that severe adverse events such as CRVO alter the treatment protocols of oncologists, which may influence the prognosis of systemic malignancy. Most patients in previous literature and in our case series were otherwise healthy, with normal lab results and well-controlled or no systemic comorbidities. Particularly, Li et al. (17) reported a 42-year-old man with no comorbidities. Jenkins et al. (20) reported bilateral hemorrhagic and ischemic retinopathy in a 62-year-old woman who was on a regular regimen of axitinib, another kind of TKI, for metastatic RCC. She was otherwise healthy, with no systemic or ocular comorbidities. Her retinal condition significantly improved with the cessation of axitinib without ocular treatment. Other reported cases mostly had well-controlled comorbidities such as HTN and hyperlipidemia. For these patients, RVO could hardly be explained other than the use of systemic TKIs, despite the presence of primary malignancies.

In addition to apatinib, camrelizumab, a programmed cell death receptor 1 (PD-1) inhibitor, was used in combination with our third patient. A synergistic antitumor effect of immunotherapy and anti-angiogenesis is well-recognized and often used as neoadjuvant therapy (44). Up to date, only one case of CRVO was reported with anti-PD-1 therapy (45), which is rarer than TKI. Although the possibility of camrelizumab-induced CRVO cannot be excluded, it is likely that apatinib contributes more to the disease onset in this patient.

All our patients developed CRVO during the use of systemic anti-VEGF agents, namely TKIs. The initial treatment of the third patient was local anti-VEGF agents, but the ME did not resolve, compromising faith in intravitreal anti-VEGF agents. On the other side, the poor responses to anti-VEGF agents indicate that TKIs might be the trigger of the retinal toxic reactions, resulting in CRVO. In comparison, IDI demonstrated good efficacy as the initial treatment or after the regimen switch, implicating the role of inflammation over VEGF in the pathogenesis of TKI-induced CRVO. In the setting of retinal endothelial injuries that might be related to the blockade of VEGF signaling pathways by systemic TKIs, IDI may be preferred in future patients with similar presentations.

Our study had the inherent limitations of a case series with a small sample size. The exact mechanism of the retinal toxicity of TKIs cannot be elucidated at this point, and the contribution of malignancy to CRVO is hard to evaluate. In addition, we lacked a control group to compare the efficacy of anti-VEGF agents and IDIs. However, to the best of our knowledge, this may be the first review on the topic of CRVO related to the use of systemic TKIs. Since the condition is rare, joint efforts should be made to reveal the incidence of CRVO and retinal toxicities in patients on systemic TKIs for metastatic malignancies.

## Conclusion

In this case series report, all patients diagnosed with CRVO on TKIs received dexamethasone implant treatment and obtained a fluid-free macula until the last visits, indicating intravitreal injection of IDIs may be preferred over anti-VEGF agents. Oncologists should be aware of the possibility of CRVO in patients on TKIs for metastatic malignancies and inform them of the potential retinal toxicity. Regular screening by a retinal specialist is necessary.

## Author contributions

ML: Data curation, Formal analysis, Methodology, Writing – original draft. LS: Data curation, Writing – original draft. RD: Writing – review & editing. YC: Writing – review & editing. CW: Conceptualization, Data curation, Funding acquisition, Supervision, Writing – review & editing.
